# Population genetic structure and colonization history of short ninespine sticklebacks (*Pungitius kaibarae*)

**DOI:** 10.1002/ece3.1594

**Published:** 2015-07-07

**Authors:** Han-Gyu Bae, Ho Young Suk

**Affiliations:** Department of Life Sciences, Yeungnam University280 Daehak-ro, Gyeongsan, Gyeongsangbuk-do, 712-749, Korea

**Keywords:** Biogeographic history, genetic structure, microsatellites, mtDNA, *Pungitius kaibarae*

## Abstract

The contemporary distribution and genetic structure of a freshwater fish provide insight into its historical geodispersal and geographical isolation following Quaternary climate changes. The short ninespine stickleback, *Pungitius kaibarae*, is a small gasterosteid fish occurring in freshwater systems on the Korean Peninsula and in southeast Russia. On the Korean Peninsula, *P. kaibarae* populations are distributed in three geographically separated regions: the NE (northeast coast), SE (southeast coast), and a limited area in the ND (Nakdong River). In this study, we used mitochondrial loci and microsatellites to investigate the evolutionary history of *P. kaibarae* populations by assessing their pattern of genetic structure. Our analyses revealed a marked level of divergence among three regional populations, suggesting a long history of isolation following colonization, although ND individuals showed relatively higher genetic affinity to populations from SE than those from NE. The populations from NE showed a great degree of interpopulation differentiation, whereas populations from SE exhibited only weak genetic structuring. Upon robust phylogenetic analysis, *P. kaibarae* formed a monophyletic group with Russian *P. sinensis* and *P. tymensis* with strong node confidence values, indicating that *P. kaibarae* populations on the Korean Peninsula originated from the southward migration of its ancestral lineage around the middle Pleistocene.

## Introduction

The contemporary distribution of species has been shaped by historical dispersal events following Quaternary climate fluctuation (Williams et al. 1998; Hewitt 2000, 2004; Provan and Bennett 2008). Many areas served as refugia for a variety of terrestrial species during Pleistocene glacial advances and as sources for recolonization following the retraction of glaciers (Hewitt 2000; Stewart and Lister 2001; Coyer et al. 2003; Hoarau et al. 2007). Freshwater species have also performed south- and northward expansion through the repeated cycles of glaciation (Bernatchez and Wilson 1998; Caldera and Bolnick 2008; Aldenhoven et al. 2010), and the pattern and rate of dispersal have likely been influenced by landscape structures and spatial isolation among natural river drainages (Bilton et al. 2001; Craw et al. 2007). Historical routes of dispersal might have been subjected to changing connections between drainage systems, for example, by formation of confluence in response to decreasing sea levels (Jeon and Suk 2014) or by stream capture events in headwater regions (Burridge et al. 2006; Craw et al. 2007). Analysis of the population genetic structure of a freshwater species can thus provide insights into the historical signature of geodispersal processes and geographical isolation with the complementary effects of drainage structures and geological episodes (Caldera and Bolnick 2008; Aldenhoven et al. 2010; Jeon and Suk 2014).

**Figure 1:**
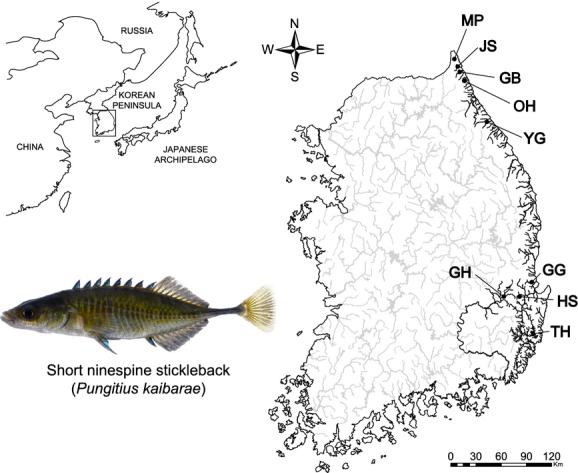
Distribution of *Pungitius kaibarae* and sampling locations. The distribution on the Korean Peninsula consisted of three regions that were geographically separated from one another, the NE (northeast coast), SE (southeast coast), and the ND (Nakdong River). The sampling sites comprised five sites on the NE, three sites on the SE, and a single site (population GH) on ND. See Table1 for detailed sampling information. The picture shows a male *P. kaibarae* caught from population GH.

The short ninespine stickleback, *Pungitius kaibarae* (Tanaka 1915), is a small-sized gasterosteid fish occurring in the freshwater system on the Korean Peninsula (Kim 1997) and in southeast Russia (Amur and Primorsky Krai; Bogutskaya et al. 2008). This species has been treated as a member of *P. sinensis* (Kobayashi 1932), as its subspecies (Chae 1988; Chae and Yang 1988; Takahashi and Goto 2001) or as a subspecies of *P. pungitius* (Okada and Matsubara 1938; Matsubara 1955). Several scientists have identified this species as distinct from other *Pungitius* species upon taxonomic reevaluation (Igarashi 1969; Kim et al. 1989). Using allozymic analyses, Yang and Min (1990) revealed a great level of genetic differentiation between *P. sinensis* and *P. kaibarae* validating them as discrete species. Although the occurrence of *P. kaibarae* populations has previously been reported in the Japanese Archipelago (Okada and Matsubara 1938), it appears that those populations have been extirpated (Miyadi et al. 1979). In the Korean Peninsula, *P. kaibarae* populations are distributed in three geographically isolated regions: the northeast coast (NE), southeast coast (SE), and a limited area (the Geumho tributary) in the Nakdong River (ND; Fig.1; Chae 1988; Chae and Yang 1988). NE and SE are separated by about 200 km, whereas the distance between SE region and Geumho tributary is just a few kilometers assuming the straight-line distance.

The Korean Peninsula is an excellent model for detecting historical events of dispersal during Quaternary climatic oscillations. This area was never completely covered by ice sheets during the multiple episodes of glaciation and therefore served as a refuge for many species (Adams and Faure 1997; Kong et al. 2014). Major rivers in the Korean Peninsula head west and south, emptying into the Yellow Sea and Korean Strait, respectively, and there are many short eastern-flowing rivers along the east coast. Western- and southern-flowing rivers have likely created confluences with the Yellow River, one of the major river systems running through China, during the climatic fluctuations of the Pleistocene epoch, whereas eastern-flowing rivers likely had confluences with the Amur River in Russia (Lindberg 1972; Nishimura 1974). Consequently, those two freshwater systems were likely exposed to very different colonization processes, which are supported by the highly distinct fauna between them (Lindberg 1972; Nishimura 1974; Kim 1997). All drainages from NE and SE regions of *P. kaibarae* distribution are east coast rivers draining east into the ocean, whereas the Nakdong River (ND region) is one of the major watersheds draining south into the ocean.

*Pungitius kaibarae* individuals from the three distinct regions display remarkable variation in the color and number of spines and the size and the number of lateral armor plates. For example, individuals from NE exhibit black-colored spines, while those from SE and the Nakdong River have blue-colored spines. Individuals from NE have a slightly greater mode number of dorsal spines (8, 7-10) than those from SE (7, 6-9; Chae 1988). In addition, individuals from NE have a similar number of armor plates across the body (31–32), whereas SE individuals show smaller-sized armor plates with great variation in number ranging from 12 to 33 (Chae 1988). Despite the geographical proximity to SE populations, individuals from the Nakdong River show a similar number of dorsal spines and armor plates to those of NE individuals (Chae and Yang 1988). A study using allozyme loci revealed considerable genetic divergence among populations from the three regions, although the population from the Nakdong River clustered more closely with SE populations (Yang and Min 1990). Kim et al. (1989) proposed that individuals from the Nakdong River originated from the Hyeungsan River in SE region by stream capture events. However, there have been no phylogenetic attempts to infer the historical dispersal and colonization of *P. kaibarae* on the Korean Peninsula to date.

Therefore, in this study, we used mitochondrial loci and microsatellites to infer the evolutionary history of *P. kaibarae* populations by assessing the pattern of genetic structure. Based on previous findings, we expected there to be strong genetic differentiation among populations from NE and SE, but not between those from SE and the Nakdong River. Given its highly limited and isolated distribution range, we also anticipated that *P. kaibarae* populations on the Korean Peninsula would be characterized by low genetic diversity. We also presented a robust phylogeny of *Pungitius* to provide insight into the historical origin of *P. kaibarae* and its geodispersal processes.

## Materials and Methods

### Sample collection

The samples used in this study were collected according to the national legislation of Republic of Korea. Adult *P. kaibarae* (6–8 cm in length) were collected from nine independent rivers from July 2012 to February 2013 using a kick net (size of the equipment: 1.2 × 0.9 m; size of stretch mesh: 10 mm). Tissue samples for genetic analyses were obtained by removing 1 × 1 mm fin clip from the tip of each tail fin, and the individuals were released back to their habitat. Populations were classified as NE (*n *=* *160 from 5 localities), SE (*n *=* *96 from 3 localities), and the Nakdong River (*n *=* *32 from a single locality) in accordance with the geographic location (Table1 and Fig.1). All of the fin clips were stored in 95% ethanol, and genomic DNA was isolated using Wizard Genomic DNA purification Kit (Promega, Madison, WI) based on manufacturer’s instructions for animal tissue with proteinase K.

**Table 1:** Sampling information of *Pungitius kaibarae* populations used for genetic analysis in the present study. The information contains geographical region, river systems, population code, location (GPS coordination), and sampling size (*n*)

Geographical region	River system	Code	GPS	*n*
NE (Northeast Coast)	Myeongpa River	MP	38° 52′ 77.37′′, 128° 39′ 81.63′′	32
	Jasan River	JS	38° 43′ 66.54′′, 128° 44′ 37.10′′	32
	Ganseong North River	GB	38° 38′ 62.50′′, 128° 45′ 82.09′′	32
	Oho River	OH	38° 32′ 04.26′′, 128° 51′ 37.13′′	32
	Yeongok River	YG	37° 84′ 70.87′′, 128° 81′ 06.73′′	32
SE (Southeast Coast)	Gokgang River	GG	36° 13′ 71.12′′, 129° 27′ 74.04′′	32
	Hyeongsan River	HS	35° 90′ 29.04′′, 129° 25′ 86.75′′	32
	Taehwa River	TH	35° 61′ 49.07′′, 129° 27′ 43.75′′	32
ND (Nakdong River)	Geumho River	GH	36° 01′ 81.08′′, 128° 97′ 93.17′′	32

### Mitochondrial DNA amplification

We used PCR (polymerase chain reaction) to amplify a partial fragment of the mitochondrial control region (CR), Cyt b (cytochrome b), and COI (cytochrome oxidase subunit I) from a total of 128 individuals in eight *P. kaibarae* populations (Table S1) using previously published primers (see Table S2 for detailed information), CR (L-Thr and H-12S; Takahashi and Goto 2001), Cyt b (PunCytBF and PunCytBR; Miya et al. 2001), and COI (HCO and LCO; Folmer et al. 1994). PCR was performed using a Piko Thermal Cycler (Finnzymes, Espoo, Finland) with a 25-*μ*L reaction mixture containing 1 *μ*L genomic DNA, 1X *Taq* buffer, 0.2 mmol/L dNTP, 0.25 *μ*mol/L of each primer, and 2.5 unit of Prime *Taq* DNA polymerase (GenetBio, Daejeon, South Korea). Thermal cycling consisted of a preliminary denaturation step at 94°C for 10 min followed by 35 cycles of 94°C for 30 sec, 54–58°C for 30 sec, and 72°C for 30 sec and final extension at 72°C for 10 min. PCR products were purified using a Primeprep PCR Purification Kit (GenetBio) for direct sequencing. The amplified products were sequenced by Genotech (Deajeon, South Korea) on an ABI 3730XL DNA Analyzer (Applied Biosystems, Foster City, CA).

### Mitochondrial DNA analysis

Sequence reads were confirmed through BLAST searches, aligned using ClustalW (Larkin et al. 2007) in MEGA 6.06 (Tamura et al. 2013), and adjusted manually when necessary. Cyt b and COI sequences were rechecked against the inferred reading frame for the corresponding protein using MEGA. All haplotypes obtained were deposited in GenBank. The genetic diversity was quantified by calculation of the number of haplotypes (*h*), haplotype diversity (*h*_d_; Nei 1987), and nucleotide diversity (*π*; Nei 1987) using DnaSP 5.10 (Librado and Rozas 2009). Tajima’s *D* and Fu’s *Fs* were estimated to test whether our data deviated from the expected values under the neutral theory model with constant population size using DnaSP. Tajima’s *D* characterize discordance between observed number of polymorphic nucleotides and the expected value quantified from average pairwise nucleotide divergence in the data set (Tajima 1989). Fu’s *Fs* characterizes the discordance between number of observed haplotypes and the value expected under the observed level of diversity (Fu 1997). Significant negative values of those indices indicate demographic expansion or background selection, whereas significant positive values indicate a recent decline in population sizes or population subdivision.

The level of genetic differentiation among populations was characterized by calculation of the genetic differentiation index (Ф_ST_) of each population pair using Arlequin 3.5 (Excoffier and Lischer 2010). The significance of the estimated Ф_ST_ values was tested using 1000 permutations of the data. Analysis of molecular variance (AMOVA) was used to test the geographical effects on population structure using Arlequin, which partitions the genetic variance among regions, among populations, and within populations. To examine the intraspecific pattern of phylogenetic relationships, a haplotype network for each mitochondrial gene was constructed based on the median-joining algorithm in SplitsTree 4.13 (Huson and Bryant 2006).

Interspecific relationships and divergence age of *P. kaibarae* were inferred under a Bayesian strict-clock method and the calibrated Yule model implemented in Beast 2.1.3 (Bouckaert et al. 2014). All other priors and options were used at the default settings. Threespine stickleback (*Gasterosteus aculeatus*; AB054361, Takahashi and Goto 2001) and brook stickleback (*Culaea inconstans*; AB445125, Kawahara et al. 2009) were used as outgroups. Although phylogenetic analysis was performed for all three mitochondrial loci we examined, only CR sequences were available for the comprehensive comparison among East Asian *Pungitius* species (NC_014889, Hwang et al. 2012; AB054320 – AB054360, Takahashi and Goto 2001). GTR+I was selected as the best nucleotide substitution model for each of the three genes under the Akaike information criterion (Akaike 1974) implemented in jModeltest 2.1.4 (Darriba et al. 2012). Two calibrations were used to estimate the divergence age. The primary calibration point was 13.3 Mya, representing the minimum age of divergence between ninespine sticklebacks (*Pungitius*) + brook sticklebacks and threespine sticklebacks based on the fossil record (Bell et al. 2009). The secondary calibration point was 7.0 Mya, indicating the stem of genus *Pungitius* (Rawlinson and Bell 1982). Two independent runs of 10 million generations were performed, sampling every 1000 generations and discarding the first 10% of logged trees as the burn-in. Two independent runs were combined in LogCombiner 2.1.3 (Bouckaert et al. 2014) and the convergence of runs was inspected as whether ESS (effective sample sizes) values were significant (>200) for all priors and the posterior distribution using Tracer 1.6 (Rambaut et al. 2014). TreeAnnotator 2.1.3 (Bouckaert et al. 2014) was used to summarize the mean divergence age and the posterior probabilities of the nodes with 95% HPD (highest posterior density) in a MCC (maximum clade credibility) tree. The final tree was visualized in FigTree 1.4.0 (Rambaut 2012).

### Microsatellite amplification

A total of 288 individuals from nine populations (Tables1 and 2) were genotyped at twelve microsatellite loci previously developed by Koizumi et al. (2007) and Meguro et al. (2009). Forward primers were fluorescently labeled with FAM, PET, VIC, or NED (Applied Biosystems). PCR conditions were the same as described in Mitochondrial DNA amplification. For each primer set, PCR amplification was conducted with annealing at 56°C. The fluorescently labeled PCR products were genotyped on an ABI 3730xl Genetic Analyzer (Biomedic, Bucheon, South Korea) and scored using GeneMapper 3.7 (Applied Biosystems).

**Table 2:** Genetic diversity of each *Pungitius kaibarae* population based on microsatellite genotyping data. Data comprises number of alleles (*A*), allelic richness (*A*_R_), inbreeding coefficient (*F*_IS_), observed heterozygosity (*H*_O_), expected heterozygosity (*H*_E_), and *P* values for the HWE (Hardy–Weinberg equilibrium) test. The expected proportion of family relationship for each population was determined by ML-Relate and Colony

Populations		*n*	*A*	*A*_R_	*F*_IS_	*H*_O_	*H*_E_	HWE	Relatedness	
									ML-Relate	Colony
NE	MP	32	5.083	5.023	0.168	0.438	0.525	<0.001	3.63	2.82
	JS	32	4.500	4.436	0.018	0.488	0.495	0.704	5.46	3.23
	GB	32	5.667	5.653	−0.017	0.531	0.522	<0.001	5.66	3.63
	OH	32	3.417	3.395	−0.043	0.482	0.462	0.317	9.88	7.86
	YG	32	3.333	3.317	−0.051	0.452	0.430	0.751	11.29	4.84
SE	GG	32	4.667	4.638	−0.004	0.510	0.508	0.712	5.04	2.41
	HS	32	6.000	5.921	0.002	0.570	0.572	0.313	1.81	2.41
	TH	32	5.167	5.129	−0.029	0.612	0.595	0.978	3.83	1.81
ND	GH	32	3.167	3.132	0.069	0.251	0.269	0.800	12.7	7.05

### Microsatellite analysis

MicroChecker 2.2.3 (Van Oosterhout et al. 2004) was used to identify the likelihood of amplification errors, that is, null alleles, large allele dropout, and stuttering. Genetic diversity was evaluated by calculating the mean number of alleles per locus (*A*) and observed (*H*_O_) and expected (*H*_E_) heterozygosities. In addition, allelic richness (*A*_R_) and fixation indices (*F*_IS_) were estimated using Arlequin and Fstat 2.9.3.2 (Goudet 2001), respectively. Deviation of genotypic proportions from those expected under HWE (Hardy–Weinberg equilibrium) was examined for each locus-population combination based on the exact test following Markov chain parameters with 1000 batches and 10,000 iterations per batch (Guo and Thompson 1992) implemented in Genepop 4.2 (Raymond and Rousset 1995). Fisher’s exact tests of linkage disequilibrium between all pairs of loci were carried out with the Markov chain algorithm in Genepop. Critical significance values were adjusted for multiple comparisons by the sequential Bonferroni correction (Rice 1989). Expected unrelated and family (full-sib and parent–offspring) relatedness for each pair of individuals within a population were determined using ML-Relate (Kalinowski et al. 2006) and Colony 2.0 (Jones and Wang 2010).

Three different approaches were used to determine whether populations may have undergone significant declines in size. Bottleneck 1.2 (Piry et al. 1999) was used to test a significant excess of *H*_E_ under the mutation-drift equilibrium relative to *H*_E_ under HWE. The heterozygosity excess was tested using the Wilcoxon sign-rank test under the TPM (two phase model) with a setting of 70% single-step mutations. The second approach was to test a mode-shift away from the typical L-shaped distribution of allelic frequencies (Luikart et al. 1998). The final approach is calculation of the *M* ratio, the mean ratio of the number of alleles to the range in allele size (Garza and Williamson 2001) using Arlequin. During a population decline, rare alleles are lost quickly, reducing the number of alleles faster than the allele size range and leading to a decrease in *M* (Garza and Williamson 2001). Critical M (Garza and Williamson 2001) was used to generate a critical value of *M* (*M*_c_) for each population. An *M* below *M*_c_ of a certain population indicates that the population has experienced a significant decline in size (Garza and Williamson 2001). *M*_c_ was calculated for two different values of *θ* (= 4*N*_e_*μ*; 1 and 10) representing *N*_e_ of 500 and 5000, respectively, with constant mutation rate of 5 × 10^−4^ and default values of Δ_g_ (the mean size of non-one-step changes; 3.5) and *P*_s_ (0.9).

Measures of multilocus differentiation among populations were calculated based on the difference in allele frequency (pairwise-*F*_ST_) and variance of allele sizes (pairwise-*R*_ST_), while testing their significance by Fisher’s exact tests after 10,000 permutations in Arlequin. A randomization (20,000 permutations) procedure was used to evaluate whether stepwise mutation has contributed to population divergence (Hardy et al. 2003) using SPAGeDi 1.5 (Hardy and Vekemans 2002). Pairwise-*D*_est_ (Jost 2008) was also calculated using SMOGD (Software for the measurement of genetic diversity; Crawford 2010) in an attempt to overcome the myth coming from the low genetic variability in several populations. The existence of distinct genetic clusters was examined in a model-based Bayesian framework implemented in Structure 2.3.4 (Pritchard et al. 2000). This analysis was performed assuming the admixture model for clusters (*K*) ranging from one to nine, with ten independent MCMC runs each consisting of 4 × 10^5^ iterations after a burn-in of 10^5^ iterations made for each *K*. The most reliable number of distinct genetic clusters was inferred using the delta *K* method described by Evanno et al. (2005) implemented in Structure Harvester 0.6.94 (Earl and vonHoldt 2012). CLUMP 1.1.2 (Jakobsson and Rosenberg 2007) was used to quantify the symmetric similarity coefficient (H′) (Nordborg et al. 2005) between pairs of runs (1000 random input sequence) with the Greedy algorithm, confirming the consistency of outcomes among ten independent runs, and to integrate the results for the selected value of *K*. Hierarchical structuring identified upon structure analysis was also tested by estimating the relative contributions of among clusters (or regions), among populations and within populations using AMOVA in Arlequin.

## Results

### Mitochondrial genetic diversity

The CR sequence consisted of 854 nucleotides with one insertion/deletion at nucleotide 206. The edited alignment contained 15 unique haplotypes (GenBank Accession Nos. KP265838 – KP265852) and 45 polymorphic sites, 42 of which could be regarded as parsimoniously informative. The mean haplotype diversity and mean nucleotide diversity of CR sequences revealed *H*_d_ = 0.817 ± 0.021 and *π *= 0.0234 ± 0.0003, respectively. All diversity estimates of CR were relatively higher in NE than in SE or population GH (ND region), although only a single haplotype was observed in the two NE populations (GB and YG; Table S1). Tajima’s *D* and Fu’s *Fs* tests of deviation from neutrality with CR sequences were only found to be significant for region SE (Table S3).

The 971 bp edited sequence alignment of Cyt b yielded 12 unique haplotypes (GenBank Accession Nos. KP265826 – KP265837) and 57 polymorphic and parsimoniously informative sites. The haplotype and nucleotide diversity of Cyt b sequences were 0.907 ± 0.008 and 0.0269 ± 0.0003, respectively. In Cyt b, NE and SE exhibited similar levels of diversity (Table S1). No significance was found upon analysis with Tajima’s *D* and Fu’s *Fs* tests of deviation from neutrality with Cyt b sequences (Table S3). The sequence alignment of 643-bp COI contained 13 unique haplotypes (GenBank Accession Nos. KM246764 – KM246776) and 48 polymorphic sites with 40 parsimoniously informative sites among them. The haplotype and nucleotide diversities of COI were 0.817 ± 0.023 and 0.0309 ± 0.0004, respectively. As with the CR locus, all diversity estimates of COI were relatively higher in NE than in SE or population GH (Table S1). Tests of deviation from neutrality in COI were only significant in the region SE for Tajima’s *D,* while no significant deviations were observed for Fu’s *Fs* (Table S3).

### Microsatellite genetic diversity

Among a total of 288 individuals, extensive polymorphism was observed at the 12 microsatellite loci examined (Tables2 and S4). The total number of alleles per locus ranged from 5 (*Omono12*) to 41 (*Ppu1*), while the observed and expected heterozygosity ranged from 0.160 (*Omono12*) to 0.858 (*Ppu1*) and from 0.164 (*Omono12*) to 0.804 (*Ppu1*), respectively. *Ppu2* was found to be out of HWE after Bonferroni adjustment (*n *=* *12, *α *= 0.0042), and *Ppu3* was out of HWE when considering *α *= 0.05 owing to significant heterozygote deficiency (Table S4). At the population level, significant deviation from the expectation was found in population MP (*n *=* *9, *α *= 0.0056; Table2). However, locus *Ppu2* and population MP were included in the further analyses, because only two population-locus combinations (*Ppu2* in MP and GB; *P* < 0.001) showed significant heterozygote deficiencies when considering pairwise comparisons of populations and loci (*n *=* *108, *α *= 0.0005). At *α *= 0.05, three population-locus combinations additionally showed significant heterozygote deficiencies (*Ppu10*-OH, *P *=* *0.027; *Omono16*-JS, *P *=* *0.029; *Ppu6*-HS, *P *=* *0.043). Fisher’s exact test showed the evidence of significant linkage disequilibrium from a single pair (*Ppu1-Omono24*, *P *<* *0.0001) after sequential Bonferroni correction (*n *=* *66, *α *= 0.0008) and revealed significant disequilibrium from three pairs (*omono7-omono8*, *P *=* *0.006; *omono4-omono16*, *P *=* *0.021; *omono12-ppu2*, *P *=* *0.001) when considering *α *= 0.05.

**Figure 2:**
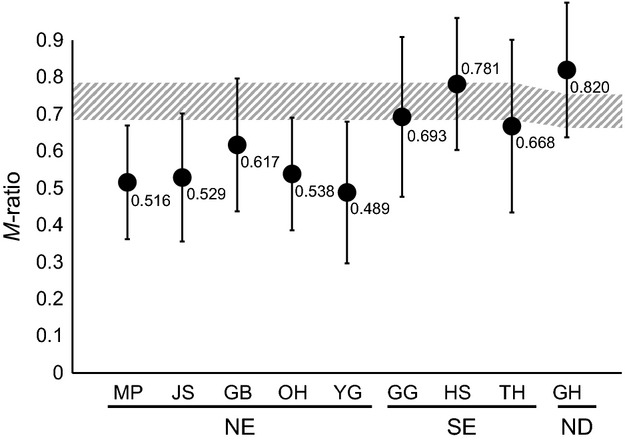
*M* ratio calculated from microsatellite genotyping. Each circle indicates the average *M* ratio of each population of *Pungitius kaibarae* with standard deviation. The dashed area indicates the *M*_c_ threshold calculated from parameter *θ *= 1 to *θ *= 10.

Microsatellite diversity estimates were highest in population HS (in *A* and *A*_R_) and TH (*H*_O_ and *H*_E_), while they were relatively low in populations OH, YG, and GH (Table2). At the regional scale, SE showed relatively higher diversity estimates than NE, but this difference was not significant (1000 permutation test in Fstat; *A*: *P *=* *0.247; *A*_R_: *P *=* *0.285). Upon maximum likelihood estimation of relatedness by ML-Relate, a low level of family relationships (1.81–11.29%) was exhibited consistently across the populations examined (Table2). The proportion of full-sib relationship estimated by Colony was also low across all populations, with values of 1.81–7.86% (Table2). The bottleneck test did not reveal any significant evidence of genetic bottleneck based on Wilcoxon’s heterozygosity excess test with TPM following Bonferroni adjustment (*n *=* *9, *α *= 0.0056) and showed evidence of bottleneck in populations OH and GH at *α *= 0.05 (Table S5). Furthermore, no evidence of population bottleneck could be inferred from the test of mode-shift in allele class distribution mode (Table S5). The signature of population size reduction was detected by *M* ratio test. Values of the *M* ratio of NE populations varied between 0.489 and 0.617, which were less than the values of *M*_c_ for each population as well as 0.68 a criterion proposed to indicate bottlenecked populations by Garza and Williamson (2001) for showing bottlenecked populations (Fig.2). By contrast, SE populations and population GH exhibited relatively larger average *M* ratios ranging from 0.668 to 0.820, which were similar to or even higher than the *M*_c_ values (Fig.2).

### Mitochondrial genetic structure

**Figure 3:**
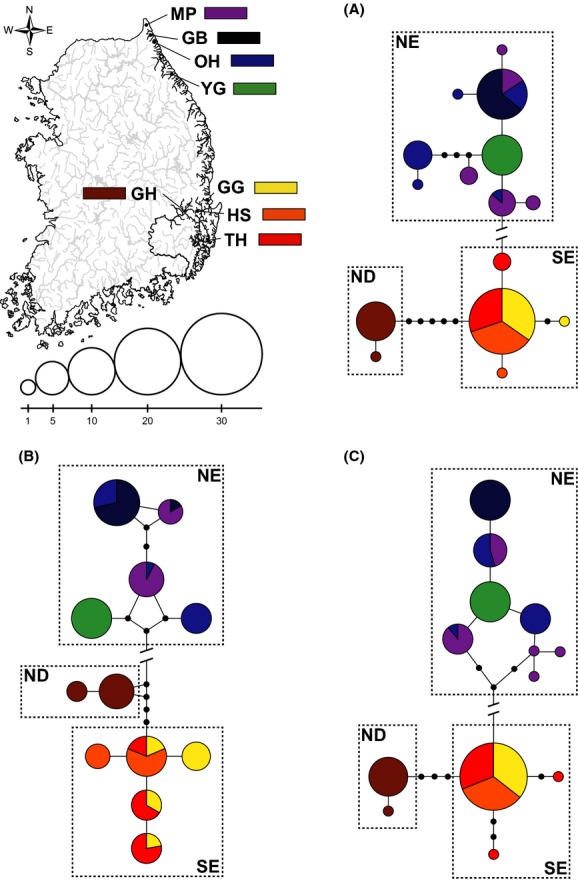
Median-joining haplotype networks of *Pungitius kaibarae*. Haplotype networks were constructed based on 854 bp of control region sequences (A), 971 bp of cytb sequences (B) and 634 bp of cytochrome oxidase subunit I sequences (C). Each circle represents a different haplotype, with its size proportional to the number of individuals found with that haplotype. The sampling sites are indicated by different colors. The indications of line-cut between NE and SE-ND represent the mutational steps of 33 (A), 47 (B) and 33 (C).

Most of the pairwise comparisons in Ф_ST_ were significant, and the overall genetic differentiations were substantial between populations from three different regions with average Ф_ST_ values of 0.975 (CR: 0.946–0.998), 0.966 (Cyt b: 0.843–0.996), and 0.983 (COI: 0.914–1.000) being observed (Table S6). Populations from different regions did not share any common haplotypes (see also Fig.3). The NE showed high population subdivision (CR: 0.326–1.000; Cyt b: 0.246–0.988; COI: 0.157–1.000), whereas populations within SE were less differentiated (CR: 0–0.100; Cyt b: 0.343–0.585; COI: −0.004 to 0; Table S6). Median-joining networks displayed for each locus revealed a strong association between haplotype variation and geographic distribution. Two completely distinct clusters, NE and SE, were observed from all networks obtained using three mitochondrial loci (Fig.3). The numbers of mutational steps between two clusters were 33 (CR), 47 (Cyt b), and 33 (COI), which were substantial (Fig.3). Haplotypes from population GH were consistently separated from those of SE populations (Fig.3). These results were confirmed by Hierarchical AMOVA results, which also revealed that most of the genetic variance was observed among regions, while only a small amount occurred among populations within regions or among individuals within populations (Table S7).

**Figure 4:**
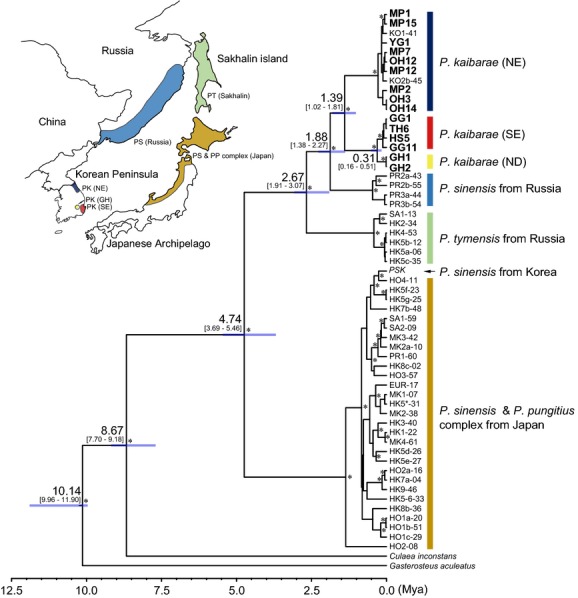
Dated Bayesian tree of genus *Pungitius* reconstructed by BEAST. Phylogeny was reconstructed with fifteen control region (CR) haplotype sequences from *Pungitius kaibarae* populations on the Korean Peninsula (denoted with bold letter) and CR haplotype sequences of *P. pungitius*, *P sinensis,* and *P. tymensis* retrieved from a study conducted by Takahashi and Goto (2001). *Gasterosteus aculeatus* (Takahashi and Goto 2001) and *Culaea inconstans* (Kawahara et al. 2009) were included as outgroups. GTR + I was used as the best-fit substitution model. Estimated divergence times from the most recent common ancestor are indicated on every major node with the 95% CI values in bracket. High posterior probabilities (>0.9) were symbolized with asterisk on the node. The distribution of different lineages or species was indicated by different colors and acronyms (PP, *Pungitius pungitius*; PS, *Pungitius sinensis*; PT, *Pungitius tymensis*; PK, *Pungitius kaibarae*) on the map.

All of the dated Bayesian trees reconstructed using the haplotypes obtained from CR, Cyt b, and COI sequence analyses consistently recovered similar grouping patterns to those shown in the median-joining network analyses with strong node confidence values. Haplotypes formed two major clusters, NE and SE + GH (Figs.4, S1 and S2). The CR data provided a comprehensive tree with many other East Asian *Pungitius* species to infer the divergence time estimation of *P. kaibarae* on the Korean Peninsula and among its phylogenetic clusters (Fig.4). Fifteen CR haplotypes of *P. kaibarae* were resolved as the most likely sister group of Russian *P. sinensis* (Fig.4). Haplotypes of *P. tymensis* were recovered as a sister group to the monophyly of *P. kaibarae* and Russian *P. sinensis*, with *P. tymensis* being placed at the ancestral position (Clade I in *Pungitius*; Fig.4). *P. sinensis* from the Korean Peninsula and Japanese Archipelago and *P. pungitius* from the Japanese Archipelago formed completely independent monophyletic clades (Clade II in *Pungitius*; Fig.4). The root node for *Pungitius* species and the divergence between clades I and II were estimated to be 4.74 Mya (CI: 3.69–5.46) and 2.67 Mya (1.91–3.07), respectively (Fig.4). Within clade I, age estimates of 1.88 Mya (1.38–2.27) and 1.39 Mya (1.02–1.81) were allocated to the nodes leading to the separation of *P. kaibarae* and the divergence between NE and SE, respectively (Fig.4). The divergence between ND (population GH) and SE were estimated to be 0.31 Mya (0.16–0.51).

### Microsatellite genetic structure

As with the mitochondrial loci, overall interpopulation genetic differentiation was considerable with global *F*_ST_ = 0.438 and *R*_ST_ = 0.644. *R*_ST_ values were significantly greater than mean permuted *R*_ST_ values (*pR*_ST_ = 0.322; *P *<* *0.001), suggesting a contribution of stepwise mutations to population divergence at the microsatellite loci. Significant *F*_ST_ and *R*_ST_ values were observed in most of pairwise comparisons (Table3). Pairwise *F*_ST_ and *R*_ST_ values ranged from 0.059 to 0.637 and 0.061 to 0.912, respectively, with strong differentiation observed upon of regions (Pairwise *F*_ST_: 0.420–0.650; Pairwise *R*_ST_: 0.573–0.912; Table3). The differentiation among populations varied significantly between NE and SE (Table3). NE exhibited a high level of population divergence, while populations within SE were less differentiated (Table3). Within NE, differentiation was especially apparent for population YG (in both *F*_ST_ and *R*_ST_; Table3). The pairwise *D*_est_ values showed similar pattern to those of pairwise *F*_ST_ and *R*_ST_, but the values between SE populations and the population GH were rather smaller than those with NE populations (Table3).

**Table 3:** Pairwise-*F*_ST_ (below the diagonal), -*R*_ST_ (above the diagonal) and *D*_est_ values of *Pungitius kaibarae* populations calculated from microsatellite genotyping. Populations are grouped by regional classification (NE, SE, and ND)

	NE					SE			ND
	MP	JS	GB	OH	YG	GG	HS	TH	GH
*F*_ST_/*R*_ST_									
MP		0.066	0.190	0.079	0.372	0.756	0.660	0.573	0.744
JS	0.095		0.277	0.208	0.452	0.764	0.666	0.581	0.747
GB	0.114	0.267		0.155	0.535	0.780	0.702	0.647	0.756
OH	0.234	0.347	0.199		0.422	0.781	0.703	0.626	0.771
YG	0.427	0.451	0.481	0.469		0.806	0.711	0.630	0.888
GG	0.471	0.511	0.502	0.503	0.525		0.103	0.125	0.912
HS	0.430	0.475	0.459	0.464	0.493	0.059		0.061	0.838
TH	0.420	0.463	0.452	0.459	0.481	0.068	0.060		0.766
GH	0.595	0.634	0.624	0.620	0.650	0.468	0.410	0.419	
*D*_est_									
JS	0.075								
GB	0.111	0.255							
OH	0.178	0.368	0.151						
YG	0.617	0.615	0.702	0.644					
GG	0.878	0.931	0.877	0.863	0.940				
HS	0.810	0.918	0.806	0.811	0.955	0.067			
TH	0.827	0.895	0.865	0.884	0.951	0.055	0.045		
GH	0.954	0.959	0.919	0.869	0.999	0.432	0.412	0.405	

**Figure 5:**
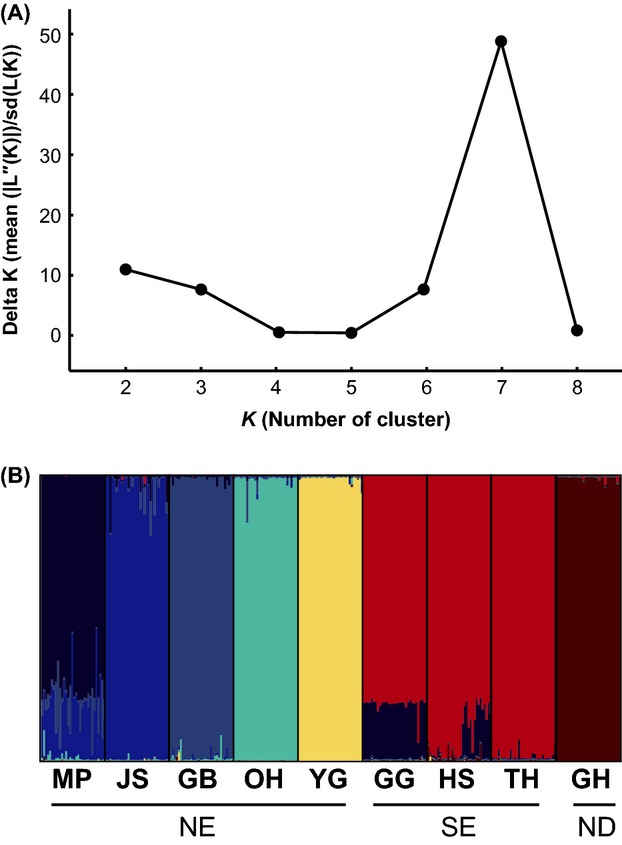
Population structure of *Pungitius kaibarae* estimated from microsatellite genotyping. The delta K method indicated that the most reliable number of cluster was seven (A). The population structure estimated in Structure 2.3.4 indicated the existence of seven genetic clusters (*K *=* *7) (B). Each cluster was denoted by different colors.

Structure analysis revealed the presence of a strong genetic structure within NE, and the delta *K* method implemented in Structure Harvester indicated an optimal clustering into seven distinct genetic clusters (Fig.5). The cluster number was also confirmed by the highest value of symmetric similarity coefficient (*H*′ = 0.832) at *K *=* *7 on CLUMPP. One cluster corresponded to SE populations including GG, HS, and TH, while the other six clusters were represented by each population (Fig.5). The results of hierarchical AMOVA showed that substantial proportion of genetic variance was found among groups characterized by structure and geographic regions compared to a small proportion among populations or within populations (Table S8).

## Discussion

### Population structure and phylogeographic history

The main objective of this study was to estimate genetic variability using nuclear and mitochondrial loci in an attempt to characterize the population structure of *Pungitius kaibarae* on the Korean Peninsula. Our genetic analyses revealed a striking level of spatial structuring in *P. kaibarae* populations corresponding to the pattern of geographic distribution, validating an earlier report based on allozymic analyses (Yang and Min 1990). At the mtDNA level, the strong genetic divergence between NE and SE was represented by the absence of shared haplotype and significant level of nucleotide differences. The 33 (CR and COI) and 47 (cyt b) mutation steps between NE and SE shown in our median-joining network analyses greatly exceeded the 95% connection limit in statistical parsimony networks generated using the method described by Clement et al. (2000), indicating a long history of allopatric divergence between the two groups following the colonization. Highly distinct allelic frequencies between NE and SE were also observed across most of the microsatellite loci examined, with an average *F*_ST_ = 0.474 and average *R*_ST_ = 0.693. In agreement with our prediction, population GH (ND region) showed relatively higher genetic affinity to populations from SE than NE, despite its genetic distinctness from all other populations.

Insight into the historical origin and geodispersal processes of *P. kaibarae* can be obtained by considering their placement within the phylogenetic tree of Gasterosteidae. While all *P. kaibarae* haplotypes detected in the present study composed a monophyletic group, Russian *P. sinensis* was resolved to be a sister taxon of *P. kaibarae*, and Russian *P. tymensis* was placed at the basal position of the Russian *P. sinensis* and *P. kaibarae* group by strong node confidence values. Thus, it appears that *P. kaibarae* was derived from the north with dispersal of its ancestral lineage. The separation age of Russian *P. sinensis* and *P. kaibarae* from *P. tymensis* represents the early Pleistocene, while the age of divergence between Russian *P. sinensis* and *P. kaibarae* was dated back to the time representing the middle Pleistocene. Around those periods, the glacial maxima formed vast areas, including the Sakhalin and the Far East Russia, tundra-like environment, which had little vegetation coverage and were unsuited for many freshwater species (Adams and Faure 1997). This likely drove ancestors of *P. kaibarae* to migrate south into the Korean Peninsula.

Based on this scenario, it is conceivable that the first founders colonized the northern part of the east coast of the Korean Peninsula and then spread south. However, no scientific evidence describing how those individuals migrated through geographically isolated drainage basins along the east coast of the peninsula has been offered. Nevertheless, one possibility of drainage connections could be envisaged on the basis of geological evidence. As the sea level fluctuated during the Pleistocene epoch (Pillans et al. 1998; Calanchi et al. 2002), the Japanese Archipelago might have been connected to the Eurasian Continent through the Korean Peninsula or Sakhalin (Keigwin and Gorbarenko 1992; Ryu et al. 2008), which likely restricted inflow of the ocean current into the East Sea (Sea of Japan) and reduced the sea surface salinity to about 20‰ (from 34‰) owing to the large amount of freshwater input from major drainage basins and precipitation (Keigwin and Gorbarenko 1992; Gorbarenko and Southon 2000; Lee and Nam 2004). Under such geological changes, *P. kaibarae* individuals might be able to migrate southward through the formation of a confluence between drainages in geographical proximity or through the sea near the coast. Considering the strong level of genetic differentiation between NE and SE, however, it is also conceivable that east coast areas have been independently colonized by two distinct founding units that were already differentiated before their dispersal. However, further study is needed to enable a more complete understanding of the colonization history of this species. Such studies include more widespread collections including sites in Russia as well as North Korea.

An earlier study (Kim et al. 1989) postulated that part of one stream in SE was captured by the Geumho River (a tributary of the Nakdong River), enabling some level of gene flow from SE populations to the Geumho River. The Geumho River is actually located close to the head of the Hyeongsan River (HS) based on a straight-line distance as short as 1–3 km. However, Yang and Min (1990) proposed that the Geumho River and SE region have been independently colonized by distinct founders based on strong allozymic divergence between SE populations and population GH. Based on the clustering pattern in the haplotype network and the phylogenetic trees, as well as the presence of shared alleles in most of the microsatellites examined, it is likely that the Geumho River has been colonized by founders that originated from SE populations, supporting the hypothesis by Kim et al. (1989). However, the overall level of genetic divergence between population GH and SE populations (e.g., average microsatellite *F*_ST_ = 0.432) may also represent the signature of isolation between the Geumho River and east coast streams. How have the three east coast stream and the Geumho River spatially been isolated following the dispersal? It is well known that the zone of Yangsan Fault is located between the Geumho River and SE region (Kyung 2010). Previous studies show that several Late Quaternary deformation events occurred in this fault zone, causing prominent landscape structure around this area (Kyung 2010). Such structure formed might have worked as barriers permanently limiting gene flow between those areas. In our data, the age of divergence between SE populations and population GH was dated back to 0.31 Mya (0.16–0.51 Mya), which is roughly consistent with the acting time (0.40 ± 0.02 and 0.42 ± 0.03 Mya) of the Yangsan Fault (Lee 2002; Kyung 2010). However, a more comprehensive resolution of the dispersal history of *P. kaibarae* into the Geumho River could be obtained by comparison with the population structure of other freshwater species inhabiting the same freshwater systems.

Asian *Pungitius* species could be subdivided into two major groups (clades I and II) in our phylogenetic analysis with CR sequences. However, a complex pattern of relationship was observed. For example, Russian *P. sinensis* did not cluster with other *P. sinensis* samples from Korea and Japan, but instead clustered more closely with *P. kaibarae* and *P. tymensis*. We suggest a comprehensive reevaluation based on morphological and genetic investigation of Asian and Russian *Pungitius* species with the background of the phylogeny obtained in the present study. First, a taxonomic revision is required for Korean *P. sinensis* and Japanese *P. sinensis* and *P. pungtius*, because those three were not clearly resolved to support separate clustering (Takata et al. 1987; Takahashi and Goto 2001; Ishikawa et al. 2013). Additionally, caution should be taken during identification of Russian *P. sinensis*. For our phylogenetic analyses, we used the haplotypes of Russian *P. sinensis* collected from the Amur and Primorsky Krai areas (Takahashi and Goto 2001). However, *P. sinensis* from Russia has never been directly compared with Korean or Japanese conspecifics. In addition, the comparison should be conducted with *P. kaibarae*, because this species was also reported in the Amur River and Primorsky Krai (Bogutskaya et al. 2008). Finally, Russian *P. kaibarae* should be directly compared with the Korean samples.

The populations from NE showed a great degree of interpopulation microsatellite differentiation, while the populations from SE exhibited weak genetic structuring. Such a pattern was visualized based on the results of Bayesian structure analysis. The streams in SE are located in geographical proximity and are not divided by complex landscape structures. It is conceivable that the parts of these adjacent streams might have been connected following major environmental events such as flooding or geological events, facilitating some level of gene flow among populations. Conversely, the streams within NE flow through successive mountain structures and are spatially separated from one another (Choi et al. 1995) with no likelihood of historical confluence. If so, the strong genetic structuring in NE could at least partially be a consequence of low levels of gene flow and the resultant small effective population sizes.

### Genetic diversity

In agreement with our prediction, a low level of mitochondrial diversity was detected, because most of the populations exhibited only a couple of haplotypes (mean number of haplotypes per population = 2.458). Population OH showed the highest mean number of haplotypes (3.667), while population YG harbored a single haplotype in all three mitochondrial loci. Haplotype diversity (*H*_d_) was slightly higher in NE than SE in CR and COI but not in Cyt b. Although the results of mitochondrial and microsatellite analyses were generally comparable, microsatellite analyses revealed a slightly different pattern in the distribution of genetic diversity, as population OH showed relatively lower levels of diversity. SE populations also showed diversity estimates slightly higher than those of NE populations. Population GH (the Nakdong River) exhibited a lower level of genetic diversity than those from NE and SE. As with the results from mitochondrial analyses, the lowest level of diversity was observed in population YG. In addition, southward expansion to the Korean Peninsula might have involved a limited number of individuals, leading to loss of genetic diversity in the founding population.

The level of mitochondrial diversity observed in our study (12–15 haplotypes from 128 individuals) is lower than those previously reported in studies with other *Pungitius* species. For example, 39 Cyt b haplotypes were found from 90 individuals of European *P. pungitius* (Shikano et al. 2010), and 97 CR haplotypes were detected from 169 individuals of North American *P. pungitius* (Aldenhoven et al., 2010). In addition, 31 CR haplotypes were found from 151 Japanese *P. pungitius* (Takahashi et al. 2003). As mentioned above, such a low level of mitochondrial diversity found in *P. kaibarae* may be attributed to its highly confined and fragmented distribution range.

Low levels of family relationship inferred from the estimation of pairwise relatedness in all populations used in our analyses. Across all populations, genetic bottlenecks were not detected using mode-shift in allele frequency distribution and heterozygote excess tests with TPM following Bonferroni adjustment, although populations OH and GH showed a significant excess of heterozygosity if considered at *α *= 0.05. However, a signature of population decline was identified across all NE populations, because they had average *M* ratios lower than the critical values for individual populations calculated by Critical M. Instead, SE populations and population GH exhibited larger average values ranging from 0.696 to 0.895. The *M* ratio is likely to indicate the signature of reduction and recovery in population size that dates back 100–300 generations (Garza and Williamson 2001; Swatdipong et al. 2010). Heterozygosity excess or allele frequency shift could only be used for the inference of genetic bottlenecks that occurred more recently (Garza and Williamson 2001). The low *M* ratios observed from five NE populations suggest that those populations have passed through a population decline that occurred maximally 200–600 years ago assuming a generation time of 2 years (Chae and Yang 1993; Swatdipong et al. 2010). This bottleneck scenario for NE populations may also be related to the strong genetic structuring within NE and drainage isolation. *P. kaibarae* is an ecological specialist preferring slow-flowing water with areas of emerging vegetation for feeding and nesting (Chae and Yang 1993). This type of habitat is less frequently found from mountainous NE streams than SE streams (Choi et al. 1995).

Although *Pungitius kaibarae* was designated as endangered in 2005 under the Protection of Wild Fauna and Flora Act of the Korean Ministry of Environment, this species was delisted in 2012 owing to the large census sizes observed throughout much of its distribution range. However, our genetic analyses have critical implications for the conservation status of this species, indicating the need for urgent management strategies. Specifically, the overall intrapopulation genetic diversity was quite low across most populations, and some of the populations exhibited a clear imprint of significant historical decline. Additionally, the distribution range of this species on the Korean Peninsula is confined to limited and geographically fragmented zones. Given that those regional groups constitute a cryptic species complex itself, the distribution should be regarded as much smaller. Each regional group of *P. kaibarae* should be considered as different ESUs (Evolutionarily Significant Units), and distinct future conservation plans should be prepared specifically for each group. Accordingly, we suggest that a comprehensive reevaluation of the conservation status of this species including the background of our genetic results be conducted.

## Supporting Information

**Figure S1.** Bayesian phylogenetic tree of genus *Pungitius* based on Cyt b sequences. **Figure S2**. Bayesian phylogenetic tree of genus *Pungitius* based on COI sequences. **Table S1**. Haplotype diversity quantified by mitochondrial CR, Cyt b and COI sequence data of *Pungitius kaibarae* populations. **Table S2**. Primer information for mitochondrial DNA analyses of *Pungitius kaibarae* populations. **Table S3**. Neutrality test based on Tajima’s *D* and Fu’s *Fs* of the three mitochondrial DNA loci performed for each population and regional group of *Pungitius kaibarae* populations. **Table S4**. Information describing 12 microsatellite loci used to genotype *Pungitius kaibarae* populations. **Table S5**. Probabilities from Wilcoxon sign-rank tests for heterozygosity excess under TPM model and examination of mode-shift in allele frequency distribution across *Pungitius kaibarae* populations. **Table S6**. Pairwise-Ф_ST_ estimates of *Pungitius kaibarae* populations based on the three mitochondrial DNA loci. **Table S7**. Hierarchical AMOVA test based on the results of three mitochondrial DNA analyses for *Pungitius kaibarae* populations. **Table S8**. Hierarchical AMOVA test based on microsatellite data of *Pungitius kaibarae* populations.

** **

** **
