# Supplementary material for: Population genetic structure and colonization history of short ninespine sticklebacks (Pungitius kaibarae)
Source: Ecol Evol. 2015 Jul 7;5(15):3075–89. doi: 10.1002/ece3.1594 (PMC4559051; doi:10.1002/ece3.1594)
Supplement: Supplementary file 1 [file ece30005-3075-sd1.docx]

**Supporting Information**

**Figure Legends**

**Figure S1** Bayesian phylogenetic tree of genus *Pungitius* based on Cyt b sequences. The values on nodes represent estimated divergence time from the most recent common ancestor with the 95% CI in bracket. High posterior probabilities (> 0.9) were symbolized by an asterisk on the node. *Gasterosteus aculeatus* (Miya *et al*., 2001) and *Culaea inconstans* (Kawahara *et al*., 2009) were used as outgroups. *P. pungitius* from the Baltic Sea (JQ983001, JQ983006 and JQ983012) (DeFaveri *et al*., 2012), *P. pungitius* from Europe (JF798873, JF798894 and JF798902) (Teacher *et al*., 2011), *P. pungitius* from Japan (NC011571) (Kawahara *et al*., 2009) and *P. sinensis* from Korea (EU332748) (Hwang *et al*., 2012) were included in the phylogenetic tree; (Citations) DeFaveri, J., Shikano, T., Ab Ghani, N.I., Merilä, J. 2012. Contrasting population structures in two sympatric fishes in the Baltic Sea basin. *Mar. Biol.* **159**: 1659-1672; Teacher, A.G.F., Shikano, T., Karjalainen, M.E., Merilä, J. 2011. Phylogeography and genetic structuring of European nine-spined sticklebacks (*Pungitius pungitius*) – mitochondrial DNA evidence. *PloS ONE.* **6**: e19476.

**Figure S2** Bayesian phylogenetic tree of genus *Pungitius* based on COI sequences. The values on nodes represent estimated divergence time from the most recent common ancestor with the 95% CI in bracket. High posterior probabilities (> 0.9) were symbolized by an asterisk on the node. *Gasterosteus aculeatus* (Miya *et al*., 2001) and *Culaea inconstans* (Kawahara *et al*., 2009) were used as outgroups. *P. Pungitius* from Japan (NC011571) (Kawahara *et al*., 2009), *P. pungitius* from Sweden (KJ128594), *P. pungitius* from Canada (KC015861) (McCusker *et al*., 2013), *P. sinensis* from Korea (EU332748) (Hwang *et al*., 2012), *Pungitius hellenicus* (KJ554514) (Geiger *et al*., 2014), *Pungitius laevis* (KJ554413) (Geiger *et al*., 2014) and *Pungitius platygaster* (KJ554378) (Geiger *et al*., 2014) were included in the phylogenetic tree; (Citations) McCusker, M.R., Denti, D., Van Guelpen, L., Kenchington, E., Bentzen, P. 2013. Barcoding Atlantic Canada's commonly encountered marine fishes. *Mol. Ecol. Resour.* **13**: 177-188; Geiger, M.F., Herder, F., Monaghan, M.T., Almada, V., Barbieri, R., Bariche, M., *et al*. 2014. Spatial heterogeneity in the Mediterranean Biodiversity Hotspot affects barcoding accuracy of its freshwater fishes*. Mol.Ecol.Resour.* **14**: 1210-1221.

**Table S1** Haplotype diversity quantified by mitochondrial CR, Cyt b and COI sequence data of *Pungitius kaibarae* populations.

| **Population** | ***n*** | **CR** | | |  | **Cyt b** | | |  | **COI** | | |
| --- | --- | --- | --- | --- | --- | --- | --- | --- | --- | --- | --- | --- |
|  |  | ***h*** | ***h_d_*** | ***π*** |  | ***h*** | ***h_d_*** | ***π*** |  | ***h*** | ***h_d_*** | ***π*** |
| **NE** | **64** | **9** | **0.765 (0.034)** | **0.0020 (0.0001)** |  | **5** | **0.778 (0.022)** | **0.0029 (0.0001)** |  | **8** | **0.818 (0.018)** | **0.0025 (0.0002)** |
| MP | 16 | 5 | 0.792 (0.064) | 0.0020 (0.0003) |  | 2 | 0.458 (0.095) | 0.0014 (0.0003) |  | 1 | - | - |
| GB | 16 | 1 | - | - |  | 2 | 0.125 (0.106) | 0.0001 (0.0001) |  | 5 | 0.683 (0.091) | 0.0030 (0.0006) |
| OH | 16 | 5 | 0.68 3(0.091) | 0.0035 (0.0004) |  | 3 | 0.575 (0.080) | 0.0026 (0.0003) |  | 3 | 0.575 (0.080) | 0.0018 (0.0003) |
| YG | 16 | 1 | - | - |  | 1 | - | - |  | 1 | - | - |
| **SE** | **48** | **4** | **0.159 (0.069)** | **0.0002 (0.0000)** |  | **5** | **0.778 (0.022)** | **0.0029 (0.0001)** |  | **3** | **0.084 (0.003)** | **0.0003 (0.0002)** |
| GG | 16 | 2 | 0.125 (0.106) | 0.0003 (0.0003) |  | 4 | 0.708 (0.091) | 0.0013 (0.0002) |  | 1 | - | - |
| HS | 16 | 2 | 0.125 (0.106) | 0.0002 (0.0001) |  | 2 | 0.500 (0.074) | 0.0005 (0.0001) |  | 1 | - | - |
| TH | 16 | 2 | 0.325 (0.125) | 0.0004 (0.0002) |  | 3 | 0.675 (0.062) | 0.0009 (0.0001) |  | 3 | 0.242 (0.135) | 0.0010 (0.0006) |
| **ND (GH)** | **16** | **2** | **0.125 (0.106)** | **0.0002 (0.0001)** |  | **2** | **0.400 (0.114)** | **0.0004 (0.0001)** |  | **2** | **0.125 (0.106)** | **0.0002 (0.0002)** |

**Table S2** Primer information for mitochondrial DNA analyses of *Pungitius kaibarae* populations. Amplification difficulty by sequence variation on primer binding sites was resolved with mixed bases used for primer design; Y is a mixture of C and T, W is a mixture of A and T, V is a mixture of G, A and C.

| **Locus** | **Primer name** | **Sequence (5′ ⟶ 3′)** | ***T*_a_ (°C)** |
| --- | --- | --- | --- |
| Control region (CR) | L-Thr | AGC TCA GCG YCA GAG CGC CGG TCT TGT AA | 58 |
|  | H-12S | TAA AGT CAG GAC CAA GCC TT |  |
| Cytochrome b (Cyt b) | PunCytBF | ATG AAA CTT TGG TTC CCT CC | 54 |
|  | PunCytBR | CGC TGA GCT ACT TTT GCA TGT |  |
| Cytochrome oxidase subunit 1 (COI) | HCO | AAA CYT CWG GGT GVC CAA AGA AYC | 54 |
|  | LCO | TAC TAA YCA CAA AGA TAT TGG CAC |  |

**Table S3** Neutrality test based on Tajima’s *D* and Fu’s *Fs* of the three mitochondrial DNA loci performed for each population and regional group of *Pungitius kaibarae* populations.

|  | **CR** | |  | **Cyt b** | |  | **COI** | |
| --- | --- | --- | --- | --- | --- | --- | --- | --- |
|  | **Tajima’s *D*** | **Fu's *Fs*** |  | **Tajima’s *D*** | **Fu's *Fs*** |  | **Tajima’s *D*** | **Fu's *Fs*** |
| **NE** | **-0.11** | **0.41** |  | **2.25** | **4.12** |  | **0.18** | **-0.90** |
| MP | 0.36 | 0.71 |  | 1.51 | 3.60 |  | 0.16 | 0.17 |
| GB | - | - |  | -1.16 | -0.70 |  | - | - |
| OH | 0.84 | 2.12 |  | 2.28 | 3.66 |  | 0.79 | 1.31 |
| YG | - | - |  | - | - |  | - | - |
| **SE** | **-1.67**** | **-2.16*** |  | **1.01** | **0.58** |  | **-1.99**** | **-1.29** |
| GG | -1.50* | 0.77 |  | 1.03 | 0.20 |  | - | - |
| HS | -1.16 | -0.70 |  | 1.31 | 1.25 |  | - | - |
| TH | 0.16 | 0.55 |  | 1.05 | 0.65 |  | -1.93** | 0.04 |
| **ND (GH)** | **-1.16** | **-0.70** |  | **0.65** | **0.87** |  | **-1.16** | **-0.70** |

**Table S4** Information describing 12 microsatellite loci used to genotype *Pungitius kaibarae* populations. The information contains repeat motifs, range of allele size, number of alleles (*A*), allelic richness (*A*_R_), *F*_ST_, *F*_IS_, *R*_ST_, observed heterozygosity (*H*_E_), expected heterozygosity (*H*_E_) and *p* values obtained from the exact test for Hardy-Weinberg equilibrium (HWE). Amplification difficulty due to sequence variations in the primer binding sites was resolved with mixed bases used for primer design; Y is a mixture of C and T, M is a mixture of A and C.

| **Locus** | **Repeat motif** | **Size range (bp)** | **Sequence (5′ ⟶ 3′)** | ***A*** | ***A*_R_** | ***F*_IS_** | ***F*_ST_** | ***R*_ST_** | ***H*_O_** | ***H*_E_** | **HWE** |
| --- | --- | --- | --- | --- | --- | --- | --- | --- | --- | --- | --- |
| Omono4 | (CA)_10_ | 108 - 124 | F: CAG CCA CAT CTG TGA GCA TC  R: TTC CGA ACA GGT GGA CTG AT | 6 | 5.549 | -0.070 | 0.773 | 0.895 | 0.184 | 0.172 | 0.812 |
| Omono7 | (CA)_3_(GA)_1_(CA)_10_(CT)_1_(CA)_3_(CT)_1_(CA)_3_ | 164 - 187 | F: GCC AGC GAA CTA TGA CCT GT  R: CGG GGT AAG CTA ATG GGT TT | 12 | 10.425 | 0.013 | 0.443 | 0.712 | 0.465 | 0.471 | 0.877 |
| Omono8 | (CA)_12_ | 152 - 178 | F: CCG TCA GTG ACT CAA CTA CCA  R: CAG CAC TCG ATT AAC CAG CA | 10 | 7.524 | 0.098 | 0.657 | 0.970 | 0.258 | 0.286 | 0.060 |
| Omono12 | (CA)_16_ | 132 - 146 | F: CAG GAA CGA GAG CCT AAC GA  R: GCC AGA TGT GCC AAG TCA TA | 5 | 4.692 | 0.023 | 0.739 | 0.874 | 0.160 | 0.164 | 0.496 |
| Omono16 | (CA)_15_ | 151 - 178 | F: CAA AGA AGC TGC TCC CTC AC  R: GCC GAT ATT GAG CGA TTA CC | 14 | 11.978 | 0.022 | 0.421 | 0.826 | 0.517 | 0.529 | 0.340 |
| Omono24 | (CA)_14_ | 108 - 188 | F: TGC CTC TGC TTT AGC TGT CA  R: CAA GGG GAT CGC ATG TAG AG | 12 | 10.707 | 0.062 | 0.270 | 0.159 | 0.579 | 0.616 | 0.326 |
| Ppu1 | (CA)_14_ | 115 - 230 | F: YGT GTT GTC ATG ACT CCA AAA C  R: CCT GGA TMA ATC TAA CCT CCT TCC | 41 | 22.919 | -0.066 | 0.165 | 0.241 | 0.858 | 0.804 | 0.967 |
| Ppu2 | (CA)_22_ | 156 - 255 | F: CAC TAC CCC TTT TGT CCA CAG  R: TGA GGT GAT TTG TAT GTG TCA GC | 24 | 13.691 | 0.147 | 0.529 | 0.763 | 0.358 | 0.423 | 0.001 |
| Ppu3 | (CA)_16_ | 184 - 230 | F: TGG TGC AAC AGG TTT TAC AGA  R: GTA TCT CCA TCA GCA CCC TTG | 17 | 13.035 | 0.036 | 0.347 | 0.752 | 0.549 | 0.569 | 0.024 |
| Ppu6 | (AC)_15_ | 191 - 215 | F: CTT YAG CCG CCT TTA ACA CG  R: GGC AGG AAA TTA GCA TGA GC | 12 | 9.970 | -0.055 | 0.346 | 0.653 | 0.616 | 0.583 | 0.960 |
| Ppu10 | (AC)_16_ | 106 - 147 | F: TGA CAT AAA CAA GAC GAT ATT CAA CA  R: CGA GAC AGT AGA AAA AGT CCA AAC TAC | 15 | 11.745 | 0.005 | 0.422 | 0.829 | 0.514 | 0.517 | 0.562 |
| Ppu13 | (CA)_28_ | 188 - 272 | F: TGA AGG GCA CAT TCA ACA YT  R: TCC AGC ACA AGG TAA GAC GA | 27 | 14.134 | -0.019 | 0.289 | 0.219 | 0.606 | 0.595 | 0.784 |

**Table S5** Probabilities from Wilcoxon sign-rank tests for heterozygosity excess under TPM model and examination of mode-shift in allele frequency distribution across *Pungitius kaibarae* populations.

| **Population** | | **Wilcoxon test (TPM)** | **Mode-shift** |
| --- | --- | --- | --- |
|  |  |  |  |
| **NE** | MP | 0.584 | Normal L shape |
|  | JS | 0.358 | Normal L shape |
|  | GB | 0.180 | Normal L shape |
|  | OH | 0.034 | Normal L shape |
|  | YG | 0.483 | Normal L shape |
|  |  |  |  |
| **SE** | GG | 0.097 | Normal L shape |
|  | HS | 0.260 | Normal L shape |
|  | TH | 0.060 | Normal L shape |
|  |  |  |  |
| **ND** | GH | 0.039 | Normal L shape |
|  |  |  |  |

**Table S6** Pairwise-Ф_ST_ estimates of *Pungitius kaibarae* populations based on the three mitochondrial DNA loci.

|  |  |  | **NE** | | | |  | **SE** | | |
| --- | --- | --- | --- | --- | --- | --- | --- | --- | --- | --- |
|  |  |  | MP | GB | OH | YG |  | GG | HS | TH |
|  |  |  |  |  |  |  |  |  |  |  |
| **CR** | **NE** | GB | 0.400 |  |  |  |  |  |  |  |
|  |  | OH | 0.326 | 0.502 |  |  |  |  |  |  |
|  |  | YG | 0.469 | 1.000 | 0.561 |  |  |  |  |  |
|  |  |  |  |  |  |  |  |  |  |  |
|  | **SE** | GG | 0.964 | 0.995 | 0.946 | 0.995 |  |  |  |  |
|  |  | HS | 0.967 | 0.998 | 0.949 | 0.998 |  | 0.000^NS^ |  |  |
|  |  | TH | 0.964 | 0.996 | 0.947 | 0.996 |  | 0.067^NS^ | 0.100^NS^ |  |
|  |  |  |  |  |  |  |  |  |  |  |
|  | **ND** | GH | 0.967 | 0.998 | 0.949 | 0.998 |  | 0.960 | 0.980 | 0.964 |
|  |  |  |  |  |  |  |  |  |  |  |
|  |  |  |  |  |  |  |  |  |  |  |
| **Cyt b** | **NE** | GB | 0.682 |  |  |  |  |  |  |  |
|  |  | OH | 0.246 | 0.558 |  |  |  |  |  |  |
|  |  | YG | 0.766 | 0.988 | 0.700 |  |  |  |  |  |
|  |  |  |  |  |  |  |  |  |  |  |
|  | **SE** | GG | 0.974 | 0.987 | 0.963 | 0.987 |  |  |  |  |
|  |  | HS | 0.981 | 0.994 | 0.969 | 0.995 |  | 0.343 |  |  |
|  |  | TH | 0.978 | 0.990 | 0.967 | 0.991 |  | 0.339 | 0.585 |  |
|  |  |  |  |  |  |  |  |  |  |  |
|  | **ND** | GH | 0.981 | 0.995 | 0.969 | 0.996 |  | 0.843 | 0.903 | 0.886 |
|  |  |  |  |  |  |  |  |  |  |  |
|  |  |  |  |  |  |  |  |  |  |  |
| **COI** | **NE** | GB | 0.648 |  |  |  |  |  |  |  |
|  |  | OH | 0.157 | 0.744 |  |  |  |  |  |  |
|  |  | YG | 0.279 | 1.000 | 0.425 |  |  |  |  |  |
|  |  |  |  |  |  |  |  |  |  |  |
|  | **SE** | GG | 0.974 | 1.000 | 0.984 | 1.000 |  |  |  |  |
|  |  | HS | 0.973 | 1.000 | 0.984 | 1.000 |  | 0.000^NS^ |  |  |
|  |  | TH | 0.965 | 0.992 | 0.976 | 0.991 |  | 0.000^NS^ | -0.004^NS^ |  |
|  |  |  |  |  |  |  |  |  |  |  |
|  | **ND** | GH | 0.974 | 0.998 | 0.984 | 0.998 |  | 0.985 | 0.984 | 0.914 |
|  |  |  |  |  |  |  |  |  |  |  |

**Table S7** Hierarchical AMOVA test based on the results of three mitochondrial DNA analyses for *Pungitius kaibarae* populations.

| **Source of variation** | **Percentage of variation** | | |
| --- | --- | --- | --- |
|  | **CR** | **Cyt b** | **COI** |
| Among regional groups | 94.41 | 93.96 | 96.46 |
| Among populations | 2.93 | 3.96 | 2.05 |
| Within populations | 2.65 | 2.08 | 1.49 |

**Table S8** Hierarchical AMOVA test based on microsatellite data of *Pungitius kaibarae* populations. *Pungitius kaibarae* populations were divided into either three (regions) or seven groups (genetic clusters from Structure analysis).

| **Source of variation** | **Percentage of variation** | |
| --- | --- | --- |
|  | **Structure** | **Regional groups** |
| Among groups | 41.37 | 33.19 |
| Among populations | 4.39 | 17.37 |
| Within populations | 0.13 | 0.12 |
| Residual | 54.10 | 49.32 |
