# Supplementary figures and images for: Population genetic structure and colonization history of short ninespine sticklebacks (Pungitius kaibarae)
Source: Ecol Evol. 2015 Jul 7;5(15):3075–89. doi: 10.1002/ece3.1594 (PMC4559051; doi:10.1002/ece3.1594)

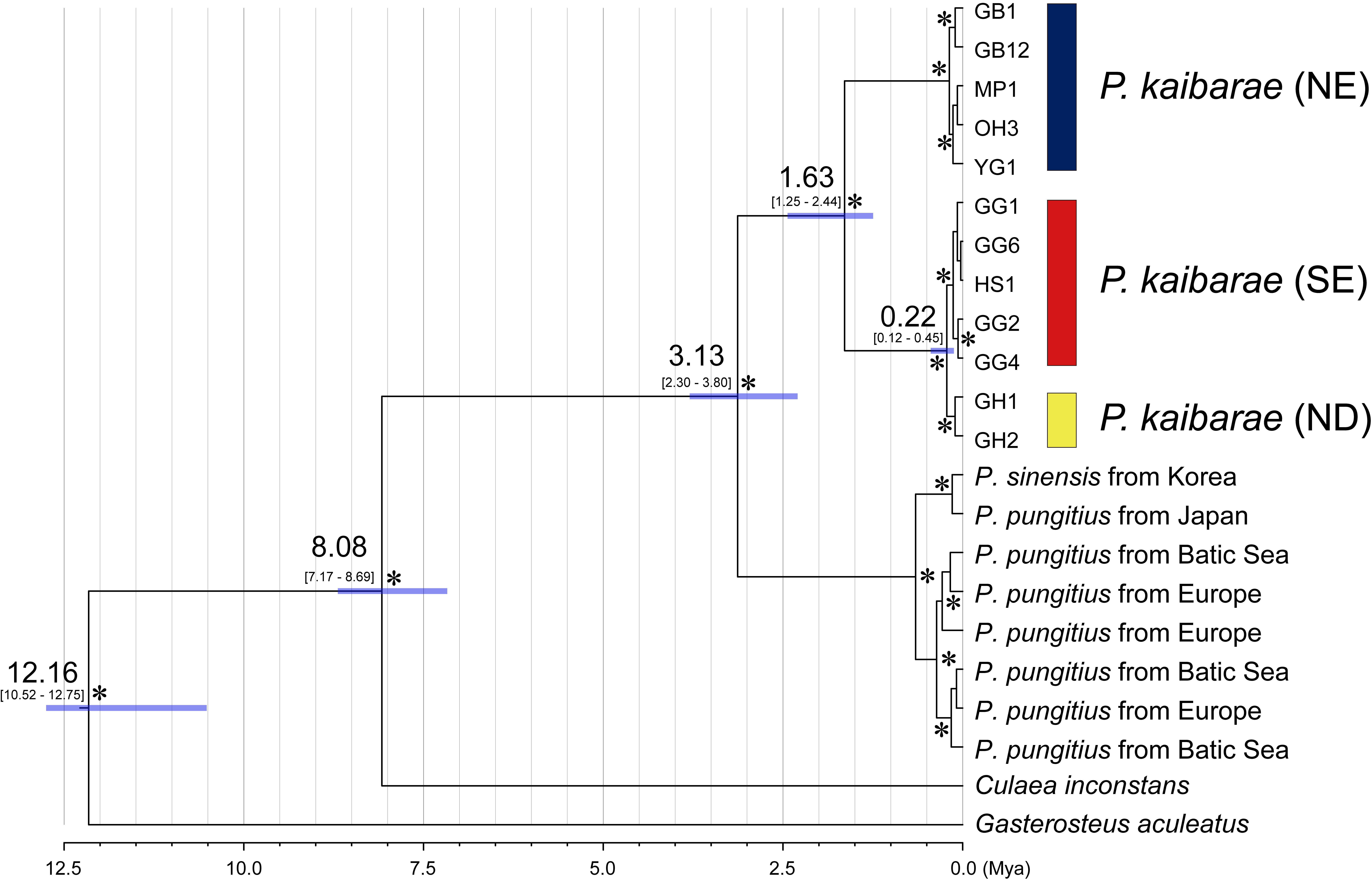

Supplement: Supplementary file 2 [file ece30005-3075-sd2.tif]

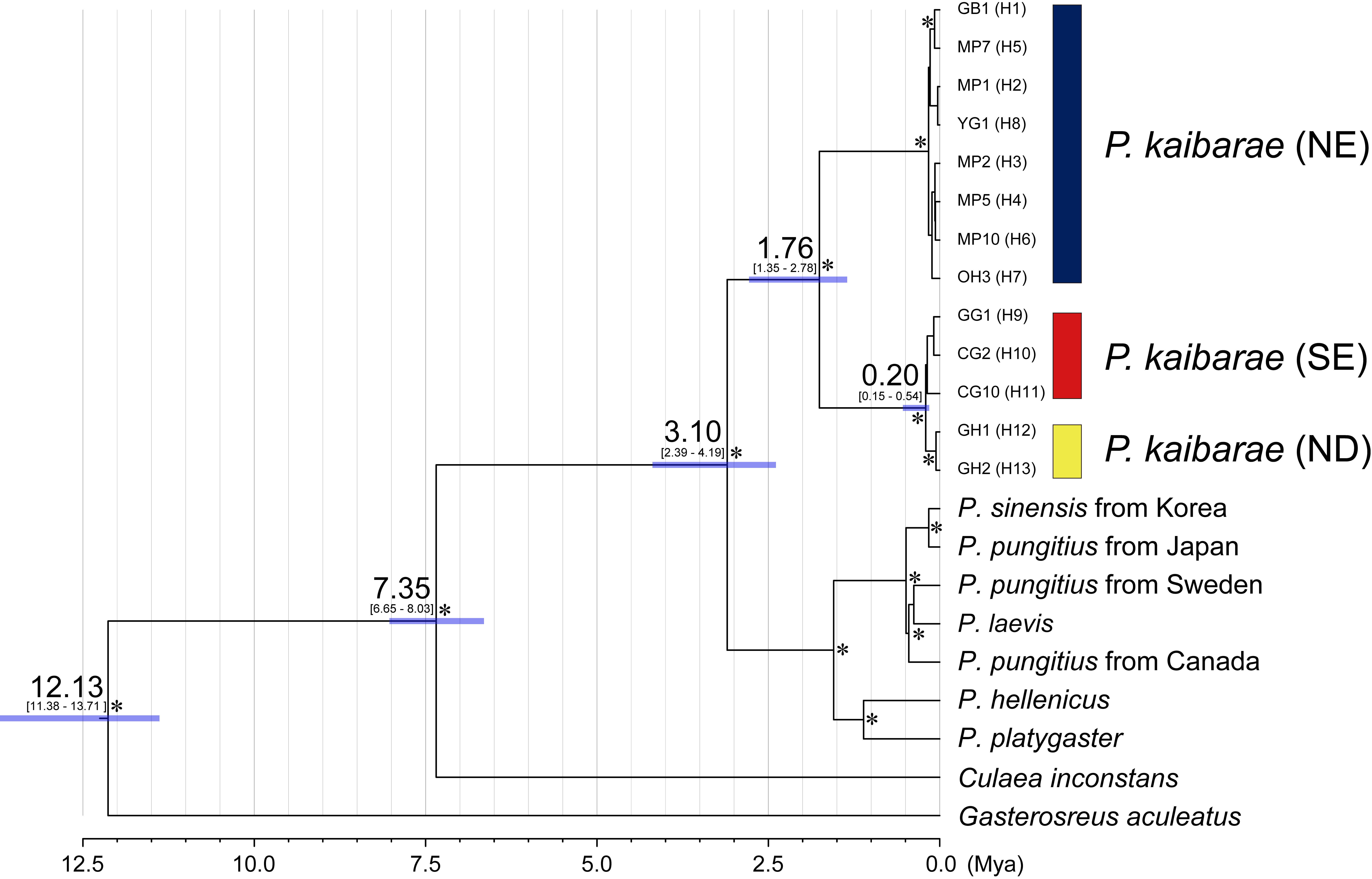

Supplement: Supplementary file 3 [file ece30005-3075-sd3.tif]
